# Lack of systematicity in research prioritisation processes — a scoping review of evidence syntheses

**DOI:** 10.1186/s13643-022-02149-2

**Published:** 2022-12-23

**Authors:** Hans Lund, Lars Tang, Ingrid Poulsen, Karen la Cour, Merete Bjerrum, Claus Vinther Nielsen, Thomas Maribo

**Affiliations:** 1 Section Evidence-Based Practice, Department of Health and Functioning, Western Norway University of Applied Sciences, 5063 Bergen, Norway; 2 The Research Unit PROgrez, Department of Physiotherapy and Occupational Therapy, Næstved-Slagelse-Ringsted Hospitals, Slagelse, Denmark; 3 Department of Regional Health Research, University of Southern Denmark, Odense, Denmark; 4 Department of Clinical Research, Copenhagen University Hospital, Amager and Hvidovre, Denmark; 5 Research Unit of Nursing and Healthcare, Department of Public Health, Aarhus University, Aarhus, Denmark; 6 Research Unit of User Perspectives and Community-Based Interventions, Department of Public Health, University of Southern Denmark, Odense, Denmark; 7 The Centre of Clinical Guidelines, Department of Clinical Medicine & The Danish Centre of Systematic Reviews — a JBI Centre of Excellence, University of Adelaide, Aalborg University, Aalborg, Denmark; 8 Department of Public Health, Aarhus University, Aarhus, Denmark; 9 DEFACTUM Central Denmark Region, Aarhus, Denmark; 10 Regionshospital Gødstrup, Herning, Denmark

**Keywords:** Evidence-based research, Research agenda, Research prioritisation process, Priority setting process, Systematicity, Transparency

## Abstract

**Background:**

A systematically and transparently prepared research priority-setting process within a specific scientific area is essential in order to develop a comprehensive and progressive evidence-based approach that will have a substantial societal impact on the site of interest. On the basis of two consensus workshops, the authors suggest the following methods for all such processes: use of experts, stakeholder involvement, literature review, and ranking.

**Objectives:**

The identification, categorisation, and discussion of methods for preparing a research prioritisation process.

**Methods:**

**Eligibility criteria:** Evidence synthesis includes original studies presenting a research prioritisation process and which listed the methods used to create a research prioritisation process. Only evidence syntheses related to health research were included.

**Data sources:** We searched the following electronic databases, without limiting by date or language: MEDLINE Ovid, Embase Ovid, Epistemonikos, and CINAHL EBSCO.

**Charting methods:** The methods used were mapped and broken down into different elements, and the use of the elements was determined. To support the mapping, (A) all of the elements were collapsed into unique categories, and (B) four essential categories were selected as crucial to a successful research prioritisation process.

**Results:**

Twelve evidence syntheses were identified, including 416 original studies. The identification and categorisation of methods used resulted in 13 unique categories of methods used to prepare a research agenda.

**Conclusion:**

None of the identified categories was used in all of the original studies. Surprisingly, all four of the essential categories were used in only one of the 416 original studies identified. There is seemingly no international consensus on which methods to use when preparing a research prioritisation process.

**Protocol registration:**

The protocol was registered in Open Science Framework (https://osf.io/dygz8/).

**Supplementary Information:**

The online version contains supplementary material available at 10.1186/s13643-022-02149-2.

## Background

The annual global investment of more than US $130 billion in health research makes it increasingly necessary to prioritise health research investment at all levels [1, 2]. Research prioritisation processes can strengthen national health research systems [3–5] and may contribute to better harmonisation of health research globally [3].

It is commonly accepted that health research priority-setting processes help researchers and policymakers to effectively target the research with the best potential to benefit public health [6, 7]. The advantages of such research prioritisation processes are the identification and resolution of clinical challenges, assistance in prioritising different research questions, the balancing of participant opinions, and the identification of knowledge gaps without influencing preferences among subgroups. However, health research prioritisation is difficult; as there are many ideas competing for research, research outcomes are inherently uncertain, and it is difficult to predict and measure what the exact impact of research will be [8]. An essential prerequisite to ensuring evidence-based practice is the conduct of the relevant and necessary research.

Previous attempts to describe research prioritisation processes have concluded that processes differ considerably in terms of methods used [1, 8, 9]. As a common standard may not be appropriate, one study prepared a checklist with nine common themes of good practice [3]. Another study highlighted four methods that combine different elements (the Essential National Health Research (ENHR), the Combined Approach Matrix (CAM), the James Lind Alliance method (JLA), and the Council on Health Research for Development (COHRED)) but emphasised that future research prioritisation processes should offer more transparency and replicability [8]. Finally, one study highlighted the waste of resources as a result of a lack of decision-making in the research prioritisation process [2].

Two systematic reviews evaluated some of the aspects of research prioritisation that we intended to identify and suggested methods to be included in the development of a research agenda [9, 10]. However, one of these reviews limited its search to studies published between 1990 and 2012 [10], and both were limited to a geographical area [9, 10]. Thus, there are no earlier studies that have systematically identified all studies conducting a research prioritisation process to determine the methods used.

Diverse methods have been used to develop a collaborative research prioritisation process, ranging from reports by expert groups to very complex processes involving hundreds of key stakeholder representatives. Thus, many different elements could potentially be included in the methods used in the research prioritisation process. At two workshops, the author team of the present study discussed the importance of these different elements and decided to produce a list of four elements that should be applied in all research prioritisation processes. The list produced included the following: (A) a systematic and transparent approach to identifying any research gaps (such as systematic reviews or scoping reviews of previous studies or a systematic search for potential earlier similar studies); (B) a systematic and transparent approach to gathering the concerns, values, preferences, experiences, and perspectives of end-users; (C) the involvement of persons with clinical and scientific expertise relevant to the planned research prioritisation process; and (D) a transparent prioritisation and consensus process among all stakeholders involved in preparing the research agenda. Systematic reviews are of vital importance. It has recently been suggested that whenever new research is planned, it should be justified on the basis of a systematic and transparent collection of current evidence from previous research, both clinical and pre-clinical. Furthermore, within a systematic and transparent approach to identifying end-users’ concerns, values, preferences, experiences, and perspectives, “end-users” are defined as those who will use and be affected by the planned research [2, 11–15].

The development of a research prioritisation process should also be based on its relevance and value for society (e.g. the burden of disease), its importance given current knowledge (e.g. evidence of the benefits of active intervention, synthesis of previous trials, and consultations with experts), the nature, scope, and severity of the problem, and a plausible explanation/rationale (preclinical research). Thus, a systematic and transparent approach that clarifies end-users’ concerns, values, preferences, experiences, and perspectives should always be part of the research prioritisation process. In addition, such a process should involve clinical and scientific experts so as to avoid impracticable, infeasible, and unrealisable project suggestions.

Finally, the suggested research prioritisation process is the result of transparency and consensus among the stakeholders in preparing this process. Hence, we intended to evaluate the extent to which these four essential elements are included in the research prioritisation process. Our explicit preconception is that all research prioritisation processes should ideally include the four essential elements. We intend to develop a systematic and transparent approach to developing research agendas that includes all relevant sources within rehabilitation research. Before embarking on such an endeavour, we need to establish an overview of the various methods that currently inform the creation of the research agenda.

This scoping review has aimed to identify, categorise, and discuss the methods used in research prioritisation processes. To accomplish this in a reasonable period of time, we decided to include evidence syntheses of studies preparing a research prioritisation process rather than original papers on developing a research prioritisation process. Therefore, on the basis of elements identified and reported in the included original studies, we were able to identify the methods used in the research prioritisation process.

## Methods

### Protocol and registration

A protocol for this scoping review (ScR) can be found at https://osf.io/dygz8/. The reporting of this scoping review follows the PRISMA guidelines extension for scoping reviews [16] (see Additional file 1).

### Procedures

The overall method consisted of initiating several workshops. First of all, a rehabilitation research group identified a need to look into research prioritisation processes due to an urgent need for an evidence-based research agenda within rehabilitation [17]. To accomplish this, the present study’s authors discussed the general issues related to the research prioritisation process at a 2-day workshop to prepare the current scoping review. At two other consensus workshops, the authors formulated the mapping of the identified methods and decided on the classification of the methods used. During these two workshops, the authors selected the four essential elements — experts (category no. 3), stakeholder involvement (category no. 7), review of literature (category no. 11), and ranking methods (category no. 12) (see Table 1) — for further use in interpreting the results of the subsequent scoping review.

**Table 1:** List of unique element categories and synonyms. For full reference to the included evidence syntheses, see Additional file 4

	Category	Explanation
0	No reported method or element	This category is presented in eight reviews (Badakhshan et al., 2018; Bryant et al., 2014; Garcia et al., 2015; Manafo et al., 2018; McGregor et al., 2014; Pii et al., 2019; Reveiz et al., 2013; Rylance et al., 2010). This category includes COHRED, descriptive study, qualitative study, road map 1, national call, not mentioned, CHNRI, pilot test, and pretest
1	Interviews	This category is presented in seven reviews (Badakhshan et al., 2018; Erntoft, 2011; Manafo et al., 2018; McGregor et al., 2014; Pii et al., 2019; Tong et al., 2015; Tong et al., 2017). This category includes interviews, in-depth interviews, semi-structured interviews, telephone interviews, key informant interviews, group interviews, interview/focus groups, pilot interviews, individual interviews, and semi-structured telephone interviews
2	Surveys	This category is presented in 10 reviews (Badakhshan et al., 2018; Booth et al., 2018; Bryant et al., 2014; Erntoft, 2011; Garcia et al., 2015; Manafo et al., 2018; McGregor et al., 2014; Pii et al., 2019; Tong et al., 2015; Tong et al., 2017). This category includes surveys, online surveys, questionnaires, web questionnaires, postal surveys, telephone surveys, the value-weighting survey method, classic weighting questionnaires, surveys of decision-making groups, surveys of research groups, surveys of funding organisations, stakeholder surveys, the listening approach, qualitative questionnaires, and interim surveys
3	Experts	This category is presented in seven reviews (Badakhshan et al., 2018; Bryant et al., 2014; Garcia et al., 2015; McGregor et al., 2014; Pii et al., 2019; Rylance et al., 2010; Tong et al., 2015). This category includes expert panels, expert forums, expert committees, expert opinions, advisory groups, expert reviews, expert research commentary, expert meetings, expert subgroups, stakeholder meetings, morphological options, advisory boards, and task forces (ENHR)
4	Workshops	This category is presented in eight reviews (Badakhshan et al., 2018; Bryant et al., 2014; Manafo et al., 2018; McGregor et al., 2014; Pii et al., 2019; Reveiz et al., 2013; Rylance et al., 2010; Tong et al., 2017). This category includes workshops, conferences/workshops, workshop consultations, working groups, working group consultations, breakout sessions, group exercises, committee meetings by conference call, and consultations
5	Focus groups	This category is presented in 11 reviews (Badakhshan et al., 2018; Booth et al., 2018; Bryant et al., 2014; Garcia et al., 2015; Manafo et al., 2018; McGregor et al., 2014; Pii et al., 2019; Reveiz et al., 2013; Rylance et al., 2010; Tong et al., 2015; Tong et al., 2017). This category includes focus groups, focus group discussions, nominal focus groups, webinars with work groups, brainstorming, nominal group techniques, case studies/community consultation models, and online forums
6	Delphi approaches	This category is presented in 10 reviews (Badakhshan et al., 2018; Booth et al., 2018; Bryant et al., 2014; Garcia et al., 2015; Manafo et al., 2018; McGregor et al., 2014; Pii et al., 2019; Reveiz et al., 2013; Tong et al., 2015; Tong et al., 2017). This category includes Delphi, the Delphi method, the modified Delphi technique, the James Lind Alliance research priority setting, and the Hanlon method
7	Stakeholder involvement	This category is presented in eight reviews (Badakhshan et al., 2018; Bryant et al., 2014; Garcia et al., 2015; Manafo et al., 2018; McGregor et al., 2014; Pii et al., 2019; Reveiz et al., 2013; Tong et al., 2017). This category includes panels with stakeholders, patient engagement panels, stakeholders, dialogue models, research needs assessment, stakeholder involvement, participation action research, deep inclusion, consumer reference group discussions, public consultations, engagement of key organisations, consultation re priorities, stakeholder roundtables, patient panels, community-based organisation partners, community advisory groups, community meetings, and feedback
8	Document analyses	This category is presented in five reviews (Bryant et al., 2014; Erntoft, 2011; Garcia et al., 2015; McGregor et al., 2014; Tong et al., 2017). This category includes environmental scans of policy issues, document analyses, analyses of a country’s situation on health, comparisons with policy, white papers, reviews of guidelines, and review/environmental scans
9	Observations	This category is presented in two reviews (Badakhshan et al., 2018; Erntoft, 2011). This category includes observations, participant observations of deliberations, observations of committee meetings, observations of deliberations
10	Consensus	This category is presented in seven reviews (Badakhshan et al., 2018; Bryant et al., 2014; Garcia et al., 2015; McGregor et al., 2014; Reveiz et al., 2013; Tong et al., 2015; Tong et al., 2017). This category includes consensus, group consensus, voting, weighting methods, rapid appraisal, roundtable discussions, go zones, public sessions, consensus metrics approaches, two meeting groups, consensus ranking, thematic analyses, workshops with the nominal group technique, interim voting, and pooling
11	Reviews of literature	This category is presented in nine reviews (Badakhshan et al., 2018; Booth et al., 2018; Bryant et al., 2014; Manafo et al., 2018; McGregor et al., 2014; Pii et al., 2019; Rylance et al., 2010; Tong et al., 2015; Tong et al., 2017). This category includes literature reviews, the James Lind Alliance research priority setting, systematic reviews, and evidence mapping
12	Ranking methods	This category is presented in eight reviews (Badakhshan et al., 2018; Garcia et al., 2015; Manafo et al., 2018; McGregor et al., 2014; Pii et al., 2019; Reveiz et al., 2013; Rylance et al., 2010; Tong et al., 2015; Tong et al., 2017). This category includes the Likert scale, weighting with the Likert scale, numerical scales, metrics, key informant ranking exercises, community consultation models, ranking, community partnered participatory research models, and discrete choice experiments
13	Conferences	This category is presented in five reviews (Garcia et al., 2015; Manafo et al., 2018; McGregor et al., 2014; Tong et al., 2015; Tong et al., 2017). This category includes meetings, seminars, retreats, teleconferences, conference calls, strategic planning meetings, multistage process meetings, and road map conferences

### Eligibility criteria

Studies satisfying the eligibility criteria were evidence syntheses (i.e. studies combining information from multiple studies investigating similar questions to come to an overall understanding of their findings). For an evidence synthesis to be deemed trustworthy, it needed, as a minimum, to include a method section explaining how the studies were identified (a systematic search strategy) and selected and how the data were extracted and either mapped or synthesised to arrive at “[a]n accurate, concise and unbiased synthesis of the available evidence” [18]. The evidence synthesis also needed to include original studies presenting a research prioritisation process within health research. Finally, the process needed to specifically identify and describe the methods applied to create a research agenda in the included original studies. No date or language limitations were involved in the search process.

### Information sources

On 4 December 2019, we searched the following electronic databases, without restricting by date or language: MEDLINE OVID (from 1946 onwards), Embase OVID (from 1947 onwards), EPISTEMONIKOS (this covers systematic reviews indexed in Cochrane, PubMed, Embase, CINAHL, PsycINFO, LILACS, Campbell Collaboration, JBI, and the EPPI-Centre Evidence Library), and CINAHL EBSCO (from 1981 onwards). We used a version of MEDLINE Ovid that contains records with the following possible statuses in addition to MEDLINE: Publisher, In-Data-Review, In-Process, and PubMed-not-MEDLINE records from NLM.

In addition, reference lists of the included evidence syntheses were used to identify other evidence syntheses potentially relevant to this scoping review.

### Search

The following search terms were used: “health priorities”, “consensus development conference”, “research agenda”, “funding priorities”, “priority setting”, “agenda setting”, and “research priorities”. These were combined with search terms for systematic reviews (i.e. “systematic review” and “scoping review”) and limited to searches in the title field (see the detailed search strategy in the appendix). No limitations were used in the search strategy. The search was prepared by TP, HL, and a librarian with expertise in searching for evidence syntheses. TP performed the final search.

### Study selection

After removing duplicates, two persons (TP, HL) independently screened the search results for inclusion and exclusion and retrieved all references selected by at least one person for further examination in full text. Title/abstract screening, full-text screening, and removal of duplicates were accomplished with the use of Rayyan [19]. The reviewers were not blinded to the journals or authors. Any discrepancies were resolved through discussion.

### Data collection process and management

All of the authors and MBR independently extracted the data using a standard data extraction form developed for this scoping review (Microsoft Excel, version 16.46). We pilot-tested the data extraction form and modified it accordingly before use. Data were extracted independently by all authors in groups of two independent reviewers. The authors resolved any discrepancies by discussing these until they reached a consensus. TM and HL quality checked all of the data and performed the analyses when the data were extracted. We extracted the following data:

- Review characteristics (author, year, journal, review type [systematic review, scoping review], number of studies included, table used for data extraction, aim/objective of the systematic review, conclusion)
- Research prioritisation process method(s) identified for each original study in the included evidence syntheses

### Mapping

**Fig. 1:**
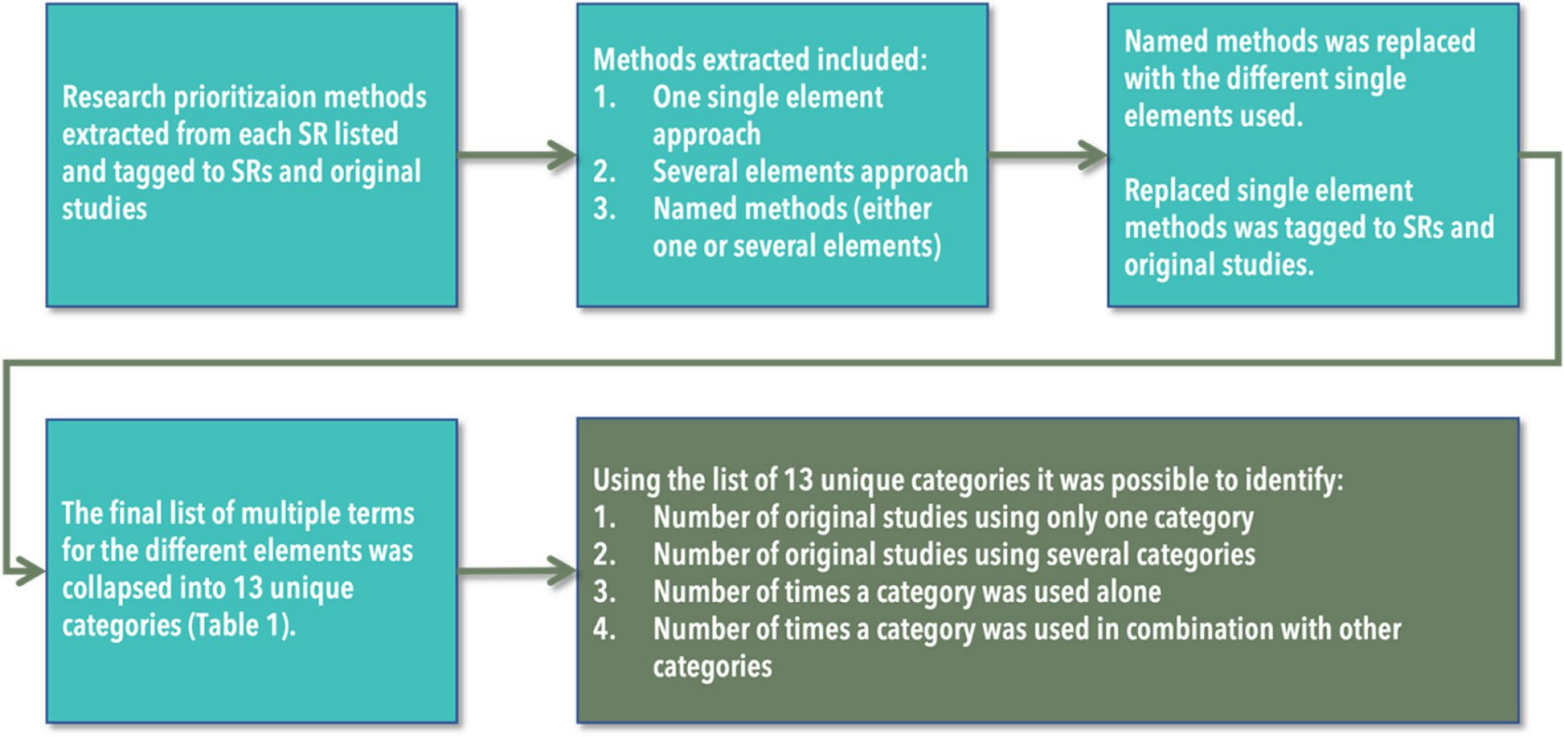
The mapping processes. SR, systematic review

All of the methods in each evidence synthesis were listed and tagged to the evidence syntheses and the included original studies. The list of methods in the included evidence syntheses consisted of (1) one single method, (2) two or more methods, and/or (3) named methods (including one or more elements) (Fig. 1). A named method includes one or more elements and has a specific name, such as CAM [9]. The exact wording of the elements used in each method was extracted from each original study included in the evidence synthesis and tagged to the systematic review and the original study from which they originated. All of the named methods were replaced with all of the single elements used and tagged to the evidence syntheses and original studies from which they originated.

The total list of elements included multiple terms for the various elements and was therefore collapsed into 13 unique element categories, where each category was exclusive and exhaustive and represented each term in that category every time the term was identified (Table 1). The categorisation was based upon a simple content analysis of the different methods referred to in the table and the text of the included evidence syntheses. This was done to ensure that the other terms could be meaningfully categorised, as in Table 1. All of the authors validated the 13 categories in two consensus workshops. Prior to each consensus workshop, HL and TM prepared suggestions for categorising all of the terms identified in the included evidence syntheses. During the workshop, all categories and review terms were discussed until consensus was reached. In some cases, debate among the co-authors led to a term being moved to another category. For the sake of clarity, all of the similar terms in each element category are presented in Table 1.

For the purposes of the study, we formulated the following questions/tasks:

1. Which categories have been used to create a research prioritisation process?
2. How often was only one category used?
3. How often were two or more categories used?
4. How often were the four essential categories combined in the same study?
5. How often were the four essential categories combined with other categories?
6. How often were the named methods used?

On the basis of the named methods referred to in similar earlier studies, the following named methods were used: the ENHR, the CAM, the James Lind Alliance method, the COHRED, and the Child Health and Nutrition Initiative (CHNRI).

## Results

**Fig. 2:**
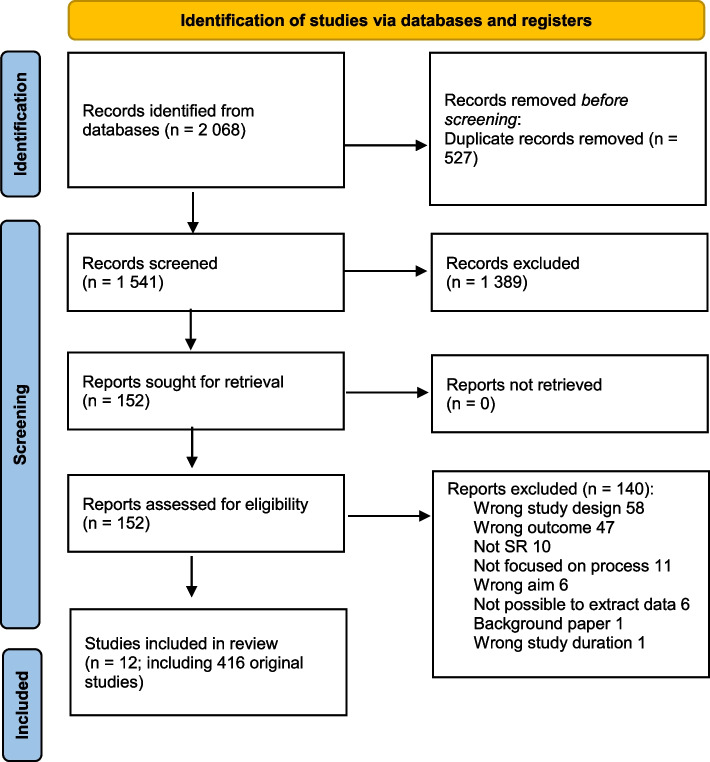
PRISMA flowchart

The database searches were conducted in November 2019 and yielded 2068 records. Following the removal of duplicates, 1541 unique records remained. After the title and abstract screening, we retrieved 152 unique evidence syntheses for full-text screening, from which we excluded 140 studies: 58 for wrong study design, 47 for wrong outcome, 11 for not describing the research prioritisation process, 10 for not being evidence syntheses, 6 for wrong aim, 6 for impossibility of extracting data, and 1 for being a background paper (Fig. 2). Twelve of the evidence syntheses met the inclusion criteria (see Table 2 and Fig. 2 for the PRISMA flowchart). A total of 416 original studies were included in these 12 evidence syntheses. One of the included reviews was referred to as a narrative review in the title [10]. As the term “narrative review” is often used as a synonym for a nonsystematic review, the study by Bryant et al. (2014) [10] should, in principle, have been excluded according to our eligibility criteria. However, Bryant et al. [10] included a method section explaining the search process (sources, search strategy, screening procedure, etc.), inclusion and exclusion criteria, and data extraction. We therefore included this study in our review.

**Table 2:** Characteristics of included studies. For full reference to the included evidence syntheses, see Additional file 4

Author and publication year	Review type	Material	Search (sources, limitations)	Overall aim	No. of included studies
Badakhshan (2018) [20]	Systematic review	Reports on priority setting in health research in the Islamic Republic of Iran	Google Scholar, PubMed, Embase, and Web of Science from inception to December 2015	Evaluating the quality of priority-setting reports on health research in the Islamic Republic of Iran	36
Booth (2018) [21]	Scoping review	Studies using any consensus consultation method	ASSIA, CINAHL, MEDLINE/MEDLINE In-Process, and Embase Searches limited to 2000 to 2017	Mapping research priorities identified in existing research prioritisation exercises regarding infants, children, and young people with life-limiting conditions	24
Bryant (2014) [10]	Narrative review	Peer-reviewed studies	MEDLINE, Cochrane, PsycINFO, and grey literature Searches limited to 1990 to March 2012	Examining methods, models, and frameworks used to make health research priorities	15
Erntoft (2011) [22]	Systematic review	Empirical articles	PubMed, Cochrane Database of Systematic Reviews, CINAHL, and PsycINFO Searches limited to 1990 to 2009	Investigating which factors and criteria are used in priority setting of pharmaceuticals	30
Garcia (2015) [23]	Systematic review	Studies in English, Spanish, or Portuguese that address the topic in the region of the Americas	Web of Science, PubMed, LILACS, and Google Searches limited to 2008 to 2013	Systematic review of literature on priorities in nursing research on health systems and services in the region of the Americas as a step towards developing a nursing research agenda	23
Manafo (2018) [24]	Rapid review	Studies conducted in Canada, the US, Europe, UK, Australia, New Zealand, and Scandinavian countries	HealthStar (via OVID), CINAHL, ProQuest databases, Scholar’s Portal Searches limited to 2001 to 2017	Describing the evidence in relation to patient and public engagement priority setting in both health ecosystems and health research	62
McGregor (2014) [9]	Systematic review	Peer-reviewed and non-peer-reviewed literature	PubMed, Embase, and CINHAL No date limits on searches; the search ended in March 2014	Priority setting processes have been undertaken in a number of low- and middle-income country (LMIC) settings and using a variety of methods. We undertook a critical review of reports of these processes	116
Pii (2019) [25]	Systematic review	Original research studies describing the involvement of cancer patients, stakeholders, and carers as active partners at any stage of the research process were included	PubMed, CINAHL, and PsycINFO Searches limited to 2006 to 2017	Describing the current state of patient and public involvement in cancer research with a focus on applied methods, among others	27
Reveiz (2013) [26]	Systematic review	National health research policies and priority agendas	The Health Research Web (HRWeb), web-based search of the official websites of health-related institutions, PubMed, LILACS, and Google Searches limited to 2002 to 2012	Comparing health research priority-setting methods and characteristics among countries in Latin America and the Caribbean between 2002 and 2012	16
Rylance (2010) [27]	Systematic review	Peer-reviewed studies related to tuberculosis	PubMed, reference lists of included studies, experts Searches limited to 1998 to 2010	Summarising existing priority statements and assessing the rigour of methods used to generate priorities for tuberculosis research	30
Tong (2017) [28]	Systematic review	Studies that directly elicited and identified research priorities for adult solid organ transplantation and published in any language in peer-reviewed journals	MEDLINE, Embase, PsycINFO from database inception, James Lind Alliance website, and Google Scholar No date limits on the searches; search ended 31 October 2016	Evaluating research priority setting in solid organ transplantation	21
Tong (2015) [29]	Systematic review	Studies that elicited patient, caregiver, healthcare provider, or policymaker priorities for research on kidney disease	MEDLINE, Embase, PsycINFO, CINAHL, reference lists of relevant articles and reviews, Google Scholar, JLA, and PubMed No date limits on searches; searches from inception to 1 May 2014	Evaluating methods to research prioritisation in kidney disease	16

The six questions

**Fig. 3:**
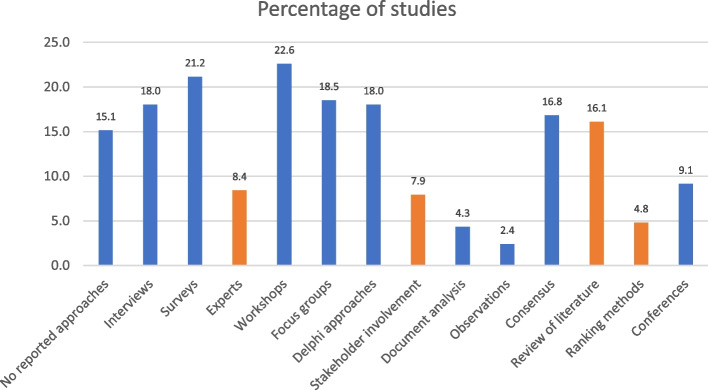
The distribution and relative use of the 13 unique categories. The orange columns illustrate the close use of the four essential categories

- Question no. 1: Which categories have been used to create a research prioritisation process? Table 1 lists the 13 unique categories of elements, and Fig. 3 illustrates the distribution of the 13 unique categories of elements taken from the 12 evidence syntheses (Table 1 and Fig. 3). Table 3 presents how often a specific category was used in each of the 12 included reviews.
- Question no. 2: How often was only one category used? On average, the 13 categories were each used 51.29 times (12.3%) in the 416 included original studies in the 12 evidence syntheses. The most frequently used element was workshops (22.6%), and the most infrequently used element was observations (2.4%).
- Question no. 3: How often were two or more categories used? Of the original studies, 42% used only one category, while 32% used two categories, and 25% used three categories or more.
- Question no. 4: How often were the four essential categories combined in the same study? Table 4 presents the combinations of the four essential categories. No combinations of three categories were identified, and only one study combined experts (no. 3), stakeholder involvement (no. 7), review of literature (no. 11), and ranking methods (no. 12) [30].
- Question no. 5: How often were the four essential categories combined with other categories? The combinations of each of the four essential categories with other categories are presented in Table 5. The most frequent combination was literature review with another category, found in 36 studies (8.65%), while 6 (1.49%) combined one of the essential categories with any other category.
- Question no. 6: How often were the named methods used? A few named methods were identified in similar earlier studies and in the included reviews and original papers (Table 6).

**Table 3:** Overview of the categories in each of the included reviews. One category may be reported more than once in an original study; thus, the number of times a category is reported may exceed the total number of included original studies in a review

	No reported approaches	Interviews	Surveys	Experts	Workshops	Focus groups	Delphi approaches	Stakeholder involvement	Document analysis	Observations	Consensus	Review of literature	Ranking methods	Conferences
Badakshan (2018) [20]	5	8	16	6	7	27	12	2	1	1	9	1	5	0
Booth (2018) [21]	0	0	2	0	0	3	20	0	0	0	0	2	0	0
Bryant (2014) [10]	3	0	11	4	4	2	1	3	3	0	2	1	0	0
Erntoft (2011) [22]	0	14	14	0	0	0	0	0	9	9	0	0	0	0
Garcia (2015) [23]	6	0	2	1	0	1	2	1	2	0	16	0	3	8
Manafo (2018) [24]	7	10	10	0	9	17	16	9	0	0	0	16	1	18
McGregor (2014) [9]	24	27	12	12	54	5	11	8	2	0	17	30	3	5
Pii (2019) [25]	3	13	11	2	4	7	4	7	0	0	0	3	2	0
Reveiz (2013) [26]	1	0	0	0	1	1	3	2	0	0	11	0	0	0
Rylance (2010) [27]	14	0	0	5	1	6	0	0	0	0	0	9	4	0
Tong (2015) [28]	0	1	2	5	0	5	4	0	0	0	4	2	1	3
Tong (2017) [29]	0	2	8	0	14	3	2	1	1	0	11	3	1	4

**Table 4:** Distribution and frequency of combinations of the four essential categories prepared by the authors

	Category no. 7 (stakeholder involvement)	Category no. 11 (review of literature)	Category no. 12 (ranking methods)
Category no. 3 (experts) combined with	4	Never	4
Category no. 7 (stakeholder involvement) combined with	-	Never	3
Category no. 11 (review of literature) combined with	Never	-	3

**Table 5:** The use of the four essential categories prepared by the authors alone or in combination with other categories (not specified). Several studies (percentage)

	Category no. 3 (experts)	Category no. 7 (stakeholder involvement)	Category no. 11 (review of literature)	Category no. 12 (ranking methods)
Category used alone (0)	2 (0.5)	11 (2.9)	11 (2.6)	1 (0.2)
Category combined with one other category	12 (2.9)	10 (2.4)	36 (8.7)	8 (1.9)
Category combined with two other categories	6 (1.5)	6 (1.5)	15 (3.6)	9 (2.2)
Category combined with three other categories	9 (2.2)	4 (1.0)	2 (0.5)	1 (0.2)
Category combined with four other categories	1 (0.2)	1 (0.2)	1 (0.2)	0
Category combined with five other categories	1 (0.2)	0	1 (0.2)	1 (0.2)

**Table 6:** The use of named methods in our material and in similar studies

Named methods	Present scoping review	Yoshida [8]	McGregor [9]	Terry [22]
Child Health and Nutrition Initiative (CHNRI)	3%	26%	18%	1%
Combined Approach Matrix (CAM)	1%	2%	-	-
Essential National Health Research (ENHR)	1%	< 1%	-	-
James Lind Alliance (JLA) method	5%	8%	-	-

## Discussion

The process yielded a list of 13 unique element categories for the methods used to prepare a research agenda. Initially, we hypothesised that four essential element categories should be included in all ideal research prioritisation process studies, while others could be added. These four essential categories were “experts” (no. 3), “stakeholder involvement” (no. 7), “literature review” (no. 11), and “ranking methods” (no. 12) (Table 1). Notably, only one study used all four of these categories [30] (included the evidence synthesis by McGregor (2014) [9]). Furthermore, the four essential categories were used in other combinations in fewer than 4% of the included original studies. The most frequently used category (review of literature, no. 11) was used in 16% of the cases, while the other three categories were used in fewer than 8% of the cases. The thirteen categories of elements identified covered a variety of elements used in the different methods reported; only two types of elements — surveys (no. 2, 21%) and workshops (no. 4, 23%) — were used in more than 20% of the cases.

We conducted this study in order to develop a framework for preparing research priority processes within rehabilitation. However, a preliminary search did not show evidence syntheses that specifically targeted rehabilitation. The methods used for preparing research priority processes in areas other than rehabilitation are thought to be similar to methods in rehabilitation. Hence, the scoping review was planned to identify and map methods for all areas of health.

Preparing a new study or a list of suggested studies (as in research agendas) without considering the existing evidence represents “a lack of scientific self-discipline that results in an inexcusable waste of public resources” (Sir Iain Chalmers’ comments [31]). Science is referred to as a cumulative enterprise right from the beginning. As Lord Rayleigh stated in 1884, “Two processes are thus at work side by side, the reception of new material and the digestion and assimilation of the old; and as both are essential, we may spare ourselves the discussion of their relative importance” (here cited from [32]). Considering that the digital revolution has fundamentally improved our present ability to “assimilate” the old studies, the ideal already set out in the seventeenth century can be expected to be apparent in any planning of new studies or research agendas. It is inexcusable that only 16% of all research prioritisation processes, according to our findings, include a literature review, as one can imagine how thrilled earlier scientists would have been had they heard of our present capabilities [33, 34]. Hence, a systematic and transparent synthesis of earlier studies with relevance to the topic of a research prioritisation process should be mandatory in all such processes, regardless of context or theme. As stated in 1984, “for science to be cumulative, an intermediate step between past and future research is necessary: synthesis of existing evidence” [35].

Furthermore, not only should a research agenda be based upon the systematic identification of research gaps, it should also systematically include the end-users’ perspectives, values, preferences, experiences, and concerns. In this context, we define end-users as those who either use the research results and/or are affected by the results. Thus, every proposal to initiate a new study should be tested to ensure its relevance to or need among end-users [11, 13] and should therefore systematically identify the prepared research agenda’s value to society [36–38]. End-users’ perspectives should be considered whenever a research prioritisation process is initiated. Category no. 7, stakeholder involvement, goes beyond end-users (including consumers, public consultations). However, only 8% of the research prioritisation processes included these groups of stakeholders in the research prioritisation process. To ensure the consideration of all critical aspects of the prioritised list of research questions in the research prioritisation process, all key stakeholders must be involved and not just end-users. This includes involving scientific and clinical experts so as to avoid the implementation of impracticable, infeasible, and unrealisable project suggestions, even though only 8% of the research prioritisation processes did so.

The preparation of a useful research agenda within a specific scientific area should include a process for prioritising agenda items. While identified research questions need to be prioritised in view of limited resources, the question is how this prioritisation should be conducted. In the list of categories (Table 1), several elements directly or indirectly include a sort of prioritisation (Delphi approaches (no. 6), consensus (no. 9), and ranking methods (no. 11)). However, in all cases, the prioritisation is based mainly on a hidden process rooted in the opinions and experiences of those invited to participate in the research prioritisation process (with JLA as an exception). Within health research, an ethical criterion could be formulated as a cornerstone of the prioritisation process: “Every major code of ethics concerning research with human subjects, from the Nuremberg Code to the present, has recognised that for clinical research to be ethically justifiable it must satisfy at least the following requirements: value, validity, and protection of the rights and welfare of participants” [36].

We acknowledge that research prioritisation processes should periodically be reviewed and updated [39], and, thus, that a transparent and systematic strategy should be applied to repeat the process. Even though several named methods were identified in this scoping review and in an earlier study [8], none of the named methods included all four of the element categories argued for here. The named methods are the ENHR, the CAM, the James Lind Alliance method, and the COHRED [1, 8].

### Earlier related studies

However, earlier studies have identified several limitations in research prioritisation processes. The studies emphasised the considerable lack of documentation of the process [1] and even of the transparency of the process [1, 2]. Two studies emphasised that most of the methods also lacked a systematic approach [1, 3], and one study showed that 78% of the processes lacked follow-up after the publication of the research agenda [9].

One study calculated how often a category was used in research priority settings in WHO [40]. In contrast to our results, Terry et al. found that 86% of the identified research prioritisation processes used experts (compared to 8.4% of the studies in our research), and 52% used literature reviews (compared to 16.1% of the studies in our research). However, the data from Terry et al. showed a very select group of studies from WHO’s technical units [40]. Another study included all of our systematic reviews and ten other reviews but made no attempt to identify how often specific categories had been used [41]. Two earlier studies reported how often a named method and three element categories were used [8, 9] (see Table 6). Another study pointed out the lack of end-user/stakeholder involvement in research prioritisation processes, and that such involvement is crucial to the research prioritisation process [1]. The involvement of end-users and other key stakeholders helps to answer questions related, for instance, to benefit, evidence, costs, efficiency, equity, equality, usefulness to a county’s economy, severity of disease, prevalence of disease, solidarity, protection of the vulnerable, and more [1]. An important topic is the relationship between the different categories. A recent study examined the importance of expert consensus versus the use of systematic reviews and showed that there is no clear answer to which is more important [42].

Several studies argued against the seeking a standard method for performing research prioritisation processes [1, 3]. According to one of the studies, “Because of the many different contexts for which priorities can be set, attempting to produce one best practice is not appropriate, as the optimal approach varies per exercise” [3]. This potentially lends support to our finding of many different methods being used. However, although context may change, certain key categories should always be included in any research prioritisation process. Hence, we suggest always including at least the four essential categories (no. 3, no. 7, no. 11, and no. 12 — see Table 1). Other items may also be considered relevant to any research prioritisation process regardless of context, such as the need to legitimise and document the process, procedures for revision and appeal, and leadership [1]. These items do not relate to identifying and prioritising research questions, but they are important prerequisites for the performance and dissemination of the final research agenda. Two studies made valuable recommendations for the entire process [3, 43], and, finally, one study argued that the whole research prioritisation process should, if possible, avoid the influence of political, economic, environmental, and idiosyncratic elements on the agenda [1]. The application of options for appeal, hearing, or revision will allow for change and adaptation with regard to different opinions [1].

Research prioritisation processes can strengthen the national health research system [3–5] and may help to better harmonise health research globally [3]. Research prioritisation processes that take a systematic, transparent approach is essential for a more transparent distribution of public and private health research funding [2, 3].

### Strengths and limitations

Almost half of the evidence syntheses limited their search in time. However, as more than 50% of the included original studies either had no time-limited search or had a search dating back to 1990, we expect the results to be unaffected by the limited searches. Our search ended in 2019, but as this study has sought to illustrate how research prioritisation processes have been performed rather to recommend how to treat patients or do research prioritisation, we have found no reason to carry out a new search. Furthermore, even though the search was performed in 2019, a later scoping review by Tan et al. (2022) conducted a search in May and June 2021 that identified several reviews, including all of the reviews included in our review. Even in this later search, Tan et al. did not identify any recent evidence syntheses that we could have included that complied with our eligibility criteria [41].

We indicated the category of ranking (no. 12) as one of four crucial categories that we argue should be used in all research prioritisation processes. However, the category of consensus (no. 10) could also include a prioritisation process; thus, 12% of the original studies had some form of ranking rather than 3% as stated (no. 12 only). The consensus process needs to be more transparent, however, and thus may not prioritise as transparently and systematically as a clear ranking process.

It was impossible to identify which end-users had been involved in the research prioritisation process, as only one review provided this information and only for two types of end-users (health professionals and patients) [25].

We did not search specifically for grey literature, as we intended to identify evidence syntheses that included all kinds of studies including grey literature. Among the 416 included original studies, reports were included that could be regarded as grey literature. For example, the only study that included all four of the essential categories prepared by the authors was a report from WHO [30].

For the sake of transparency in the collapsing of the many different elements, we have provided a detailed description of the basis for the categories in Table 1. Furthermore, as our search was comprehensive and we managed to include 416 unique original studies using earlier evidence syntheses, we have provided a highly realistic, potential picture of how research prioritisation processes within health research have been conducted over the past 25 to 30 years. Our analyses also clearly show the variation and diversity in the performance of these prioritisation processes.

### Implications for research

As we need a clearer understanding of how the different categories have been used and the reason for so much diversity in research prioritisation processes, further studies evaluating earlier prioritisation processes are needed in order to obtain further in-depth knowledge of these critical processes. An evidence synthesis covering all of the original studies and an in-depth analysis of what has occurred during the prioritisation process are thus needed. In addition, surveys and qualitative studies, including in-depth text analyses and interviews of persons involved in the research prioritisation process, would be very beneficial. Most importantly, there is a great need for studies evaluating the impact of using the essential four categories prepared by the authors. Finally, there is also a need for studies assessing the impact of including legitimisation and/or documentation of the process, procedures for revisions and appeals, and leadership in the research prioritisation process.

### Perspectives

Before mapping and analysing the results of this scoping review, we defined four essential categories for an optimal research prioritisation process regardless of the topic or context of the prioritisation process. Our results show that very few studies used one or more of these four essential categories, and only one study used all four. Even though topic and context will change, these four categories should still be used. This needs to be promoted, and the impact of using these four elements should be examined further.

## Conclusions

We have aimed primarily to establish an overview of the methods used in the performance of the research prioritisation process to develop an evidence-based research agenda within a given topic. The many different methods were collapsed into 13 categories, four of which were defined as essential — use of experts, stakeholder involvement, literature review, and the ranking of strategies — in addition to other methods. The results indicate that none of the identified categories was used in all of the original studies. Surprisingly, all four of the essential categories were used in only one of the 416 original studies identified. Thus, we conclude that there is not yet an international consensus on the preparation and prioritisation of research processes.

## Supplementary Information

**Additional file 1.** The filled-in PRISMA checklist.

**Additional file 2.** The search strategy for MEDLINE OVID, Embase OVID, and CINAHL EBSCO.

**Additional file 3.** Differences between protocol and paper.

**Additional file 4.** Reference list of the 12 included evidence syntheses.

**Additional file 5.** Reference list of all 140 excluded studies based on full-text screening.

## Data Availability

The datasets used and analysed in the current study are available from the publication website.
