# Supplementary material for: Lack of systematicity in research prioritisation processes — a scoping review of evidence syntheses
Source: Syst Rev. 2022 Dec 23;11:277. doi: 10.1186/s13643-022-02149-2 (PMC9784020; doi:10.1186/s13643-022-02149-2)
Supplement: Supplementary file 2 — Additional file 2. The search strategy for MEDLINE OVID, Embase OVID, and CINAHL EBSCO. [file 13643_2022_2149_MOESM2_ESM.docx]

Additional File 2

Search strategy

MEDLINE OVID

Embase OVID

CINAHL EBSCO

| MEDLINE | Date: 4 December 2019 |
| --- | --- |
| Agenda | SR / ScR |
| (health adj2 priorit*).ti - 1209  (consensus adj2 development adj1 conferenc*).ti - 420  (research adj2 agenda).ti - 1791  (funding adj2 priorit*).ti - 68  (priorit* adj2 setting*).ti - 1139  (agenda adj2 setting*).ti - 172  (research adj2 priorit*).ti - 1695 | (systematic adj2 review).ti - 108169  (scoping adj2 review).ti - 3315 |
| Combined | 129 |

| Embase |  |
| --- | --- |
| Agenda | SR / ScR |
| health near/2 priorit* -  (consensus NEAR/2 development adj1 conferenc*).ti -  (research NEAR/2 agenda).ti -  (funding NEAR/2 priorit*).ti -  (priorit* NEAR/2 setting*).ti -  (agenda NEAR/2 setting*).ti -  (research NEAR/2 priorit*).ti - | (systematic NEAR/2 review).ti -  (scoping NEAR/2 review).ti - |

| CINAHL |  |
| --- | --- |
| Agenda | SR / ScR |
| (health N2 priorit*).ti - 1209  (consensus N2 development adj1 conferenc*).ti -  (research N2 agenda).ti -  (funding N2 priorit*).ti -  (priorit* N2 setting*).ti -  (agenda N2 setting*).ti -  (research N2 priorit*).ti - | (systematic adj2 review).ti - 108169  (scoping N2 review).ti - |
