# Supplementary material for: Lack of systematicity in research prioritisation processes — a scoping review of evidence syntheses
Source: Syst Rev. 2022 Dec 23;11:277. doi: 10.1186/s13643-022-02149-2 (PMC9784020; doi:10.1186/s13643-022-02149-2)
Supplement: Supplementary file 3 — Additional file 3. Differences between protocol and paper. [file 13643_2022_2149_MOESM3_ESM.docx]

Additional File 3

Differences between protocol and final publication

1. Author list
Thomas Potrebny not a co-author. Due to other obligations TP had to withdraw as a co-author.

2. The research questions was adjusted

| Protocol | Final version | Comments |
| --- | --- | --- |
| 1. What kind of methods have been used to create a research agenda? | What kind of categories have been used to create a research prioritization process? | Same |
| 2. How many different processes (steps) has been included in each method? | How often has only one category been used? | Same |
| 3. How often have the different methods and different processes been used? | How often have two or more categories been used? | Same |
| 4. Would it be possible to identify some methodological archetypes used to create a research agenda? |  | 13 categories were identified – same as #1 |
|  | How often were the four essential categories combined in the same study? | In the process, the author group identified four essential categories |
| 5. Which stakeholders / end users has been involved in the research agenda building process? |  | Was not possible to answer as the included systematic reviews did not provide the data |
|  | How often were the four essential categories combined with other categories? | In the process, the author group identified four essential categories |
|  | How often were the named approaches used? Based upon the named approaches referred to in earlier similar studies, the following named approached were calculated Essential National Health Research (ENHR), Combined Approach Matrix (CAM), the James Lind Alliance Method, and Council on Health Research for Development (COHRED) and Child Health and Nutrition Initiative (CHNRI). | During the process we discovered a number of named approaches |
|  | A simple timeline for publication of the included systematic reviews and the included original studies. | Find that information useful |
| 6. How were the different stakeholders involved in the research agenda building process? |  | Was not possible to answer as the included systematic reviews did not provide the data |
|  | A timeline for the use of the four essential categories. | Find that information useful |
| 7. What sources was used to inform the research agenda building process? | What kind of categories have been used to create a research prioritization process? | The 13 categories were identified |
| 8. What kind of biases in the different procedures and choices of stakeholders and sources could be identified? |  | Was not possible to answer as the included systematic reviews did not provide the data |
|  | How often has only one category been used? | Find that information useful |
|  | How often have two or more categories been used? | Find that information useful |
