# Supplementary material for: Lack of systematicity in research prioritisation processes — a scoping review of evidence syntheses
Source: Syst Rev. 2022 Dec 23;11:277. doi: 10.1186/s13643-022-02149-2 (PMC9784020; doi:10.1186/s13643-022-02149-2)
Supplement: Supplementary file 4 — Additional file 4. Reference list of the 12 included evidence syntheses. [file 13643_2022_2149_MOESM4_ESM.docx]

Additional file 4

Reference list of the 12 included evidence syntheses

1. Badakhshan A, Arab M, Rashidian A, Gholipour M, Mohebbi E, Zendehdel K. Systematic review of priority setting studies in health research in the Islamic Republic of Iran. East Mediterr Health J. 2018;24(8):753-69.
2. Booth A, Maddison J, Wright K, Fraser L, Beresford B. Research prioritisation exercises related to the care of children and young people with life-limiting conditions, their parents and all those who care for them: A systematic scoping review. Palliat Med. 2018;32(10):1552-66.
3. Bryant J, Sanson-Fisher R, Walsh J, Stewart J. Health research priority setting in selected high income countries: a narrative review of methods used and recommendations for future practice. Cost Eff Resour Alloc. 2014;12:23.
4. Erntoft S. Pharmaceutical priority setting and the use of health economic evaluations: a systematic literature review. Value in health : the journal of the International Society for Pharmacoeconomics and Outcomes Research. 2011;14(4):587-99.
5. Garcia AB, Cassiani SH, Reveiz L. A systematic review of nursing research priorities on health system and services in the Americas. Rev Panam Salud Publica. 2015;37(3):162-71.
6. Manafo E, Petermann L, Vandall-Walker V, Mason-Lai P. Patient and public engagement in priority setting: A systematic rapid review of the literature. PLoS One. 2018;13(3):e0193579.
7. McGregor S, Henderson KJ, Kaldor JM. How are health research priorities set in low and middle income countries? A systematic review of published reports. PLoS One. 2014;9(9):e108787.
8. Pii KH, Schou LH, Piil K, Jarden M. Current trends in patient and public involvement in cancer research: A systematic review. Health expectations : an international journal of public participation in health care and health policy. 2019;22(1):3-20.
9. Reveiz L, Elias V, Terry RF, Alger J, Becerra-Posada F. Comparison of national health research priority-setting methods and characteristics in Latin America and the Caribbean, 2002-2012. Rev Panam Salud Publica. 2013;34(1):1-13.
10. Rylance J, Pai M, Lienhardt C, Garner P. Priorities for tuberculosis research: a systematic review. Lancet Infect Dis. 2010;10(12):886-92.
11. Tong A, Sautenet B, Chapman JR, Harper C, MacDonald P, Shackel N, et al. Research priority setting in organ transplantation: a systematic review. Transplant international : official journal of the European Society for Organ Transplantation. 2017;30(4):327-43.
12. Tong A, Chando S, Crowe S, Manns B, Winkelmayer WC, Hemmelgarn B, et al. Research priority setting in kidney disease: a systematic review. Am J Kidney Dis. 2015;65(5):674-83.
