# Supplementary material for: Lack of systematicity in research prioritisation processes — a scoping review of evidence syntheses
Source: Syst Rev. 2022 Dec 23;11:277. doi: 10.1186/s13643-022-02149-2 (PMC9784020; doi:10.1186/s13643-022-02149-2)
Supplement: Supplementary file 5 — Additional file 5. Reference list of all 140 excluded studies based on full-text screening. [file 13643_2022_2149_MOESM5_ESM.docx]

Additional file 5

Reference list over excluded studies (based upon full text screening)

1. Agarwal S, Kirk K, Sripad P, Bellows B, Abuya T, Warren C. Setting the global research agenda for community health systems: literature and consultative review. Human Resources for Health. 2019;17(1):N.PAG-N.PAG.
2. Akinyemi JO, De Wet N, Odimegwu CO. Review of Sexuality Studies in Africa: Setting a New Post-2015 Research Agenda. African Journal of Reproductive Health. 2016;20(1):21-8.
3. Andrews J. Agency for Healthcare Research and Quality. 2013;12(13):01.
4. Angell B, Dodd R, Palagyi A, Gadsden T, Abimbola S, Prinja S, et al. Primary health care financing interventions: a systematic review and stakeholder-driven research agenda for the Asia-Pacific region. BMJ Global Health. 2019;4:e001481.
5. Anstee S, Price A, Young A, Barnard K, Coates B, Fraser S, et al. Developing a matrix to identify and prioritise research recommendations in HIV prevention. BMC Public Health. 2011;11(1):381-.
6. Apesoa-Varano EC, Tang-Feldman Y, Reinhard SC, Choula R, Young HM. Multi-Cultural Caregiving and Caregiver Interventions: A Look Back and a Call for Future Action. Generations. 2015;39(4):39-48.
7. Aslakson RA, Reinke LF, Cox C, Kross EK, Benzo RP, Curtis JR, et al. Developing a Research Agenda for Integrating Palliative Care into Critical Care and Pulmonary Practice To Improve Patient and Family Outcomes. Journal of Palliative Medicine. 2017;20(4):329-43.
8. Averis A, Pearson A. Filling the gaps: identifying nursing research priorities through the analysis of completed systematic reviews. JBI Reports. 2003;1(3):49-126.
9. Bembea MM, Valentine SL, Bateman ST, Wilson LM, Anton B, Harger N, et al. The Pediatric Critical Care Transfusion and Anemia Expertise Initiative Consensus Conference Methodology. Pediatric Critical Care Medicine. 2018;19:S93-S7.
10. Bennett WL, Robinson KA, Saldanha IJ, Wilson LM, Nicholson W, K. a. High Priority Research Needs for Gestational Diabetes Mellitus. Journal of Women's Health (15409996). 2012;21(9):925-32.
11. Bolton LB, Donaldson NE, Rutledge DN, Bennett C, Brown DS. The impact of nursing interventions: overview of effective interventions, outcomes, measures, and priorities for future research. Medical Care Research & Review. 2007;64:123S-43S.
12. Bourne AM, Johnston RV, Cyril S, Briggs AM, Clavisi O, Duque G, et al. Scoping review of priority setting of research topics for musculoskeletal conditions. BMJ Open. 2018;8(12):e023962.
13. Bragge P, Piccenna L, Middleton JW, Williams S, Creasey G, Dunlop S, et al. Developing a spinal cord injury research strategy using a structured process of evidence review and stakeholder dialogue. Part I: rapid review of SCI prioritisation literature. Spinal Cord. 2015;53(10):714-20.
14. Bratzke LC, Muehrer RJ, Kehl KA, Kyoung Suk L, Ward EC, Kwekkeboom KL. Self-management priority setting and decision-making in adults with multimorbidity: A narrative review of literature. International Journal of Nursing Studies. 2015;52(3):744-55.
15. Bublitz MH, Stroud LR, Bublitz MH, Stroud LR. Maternal smoking during pregnancy and offspring brain structure and function: review and agenda for future research. Nicotine & Tobacco Research. 2012;14(4):388-97.
16. Buckley BS, Grant AM, Glazener CMA. Case study: a patient-clinician collaboration that identified and prioritized evidence gaps and stimulated research development. Journal of Clinical Epidemiology. 2013;66(5):483-9.
17. Butler M, Forte ML, Kane RL, Swiontkowski MF. Agency for Healthcare Research and Quality. 2010:09.
18. Byron MJ, Cohen JE, Frattaroli S, Gittelsohn J, Drope JM, Jernigan DH. Implementing smoke-free policies in low- and middle-income countries: A brief review and research agenda. Tobacco Induced Diseases. 2019;17:1-10.
19. Camelo Castillo W, Heath N, Kim J, Yang K, Ritchey ME, dosReis S, et al. Engaging stakeholders in pharmacoepidemiology research: Current state and recommendations. Pharmacoepidemiology & Drug Safety. 2019;28(6):766-76.
20. Carey TS, Crotty KA, Morrissey JP, Jonas DE, Thaker S, Ellis AR, et al. Future research needs for evaluating the integration of mental health and substance abuse treatment with primary care. Journal of Psychiatric Practice. 2013;19(5):345-59.
21. Carroll LJ, Hurwitz EL, Côté P, Hogg-Johnson S, Carragee EJ, Nordin M, et al. Research priorities and methodological implications: The Bone and Joint Decade 2000-2010 Task Force on Neck Pain and Its Associated Disorders. Spine (03622436). 2008;33:S214-20.
22. Challa S, Wu HH, Cunningham BP, Liu M, Patel K, Shearer DW, et al. Orthopaedic Trauma in the Developing World: Where Are the Gaps in Research and What Can Be Done? Journal of Orthopaedic Trauma. 2018;32:S43-S6.
23. Chang SM, Carey TS, Kato EU, Guise JM, ers GD. Identifying research needs for improving health care. Annals of Internal Medicine. 2012;157(6):439-45.
24. Chew PKH, Dillon DB. Statistics Anxiety Update: Refining the Construct and Recommendations for a New Research Agenda. Perspectives on Psychological Science. 2014;9(2):196-208.
25. Christian RB, Gaynes BN, Saavedra LM, Sheitman B, Wines R, Jonas DE, et al. Use of antipsychotic medications in pediatric and young adult populations: future research needs. Journal of Psychiatric Practice. 2015;21(1):26-36.
26. Chua PL, Dorotan MM, Sigua JA, Estanislao RD, Hashizume M, Salazar MA. Scoping Review of Climate Change and Health Research in the Philippines: A Complementary Tool in Research Agenda-Setting. International Journal of Environmental Research & Public Health [Electronic Resource]. 2019;16(14):23.
27. Clevenger CK, Chu TA, Yang Z, Hepburn KW. Clinical Care of Persons with Dementia in the Emergency Department: A Review of the Literature and Agenda for Research. Journal of the American Geriatrics Society. 2012;60(9):1742-8.
28. Coates LC, FitzGerald O, Merola JF, Smolen J, van Mens LJJ, Bertheussen H, et al. Group for Research and Assessment of Psoriasis and Psoriatic Arthritis/Outcome Measures in Rheumatology Consensus‐Based Recommendations and Research Agenda for Use of Composite Measures and Treatment Targets in Psoriatic Arthritis. Arthritis & Rheumatology. 2018;70(3):345-55.
29. Consunji RJ, Hyder AA. The burden of injuries in the Philippines: implications for national research policy. Accident Analysis & Prevention. 2004;36(6):1111-7.
30. Covell CL, Neiterman E, Bourgeault IL. A Scoping Review of the Literature on Internationally Educated Nurses in Canada: Mapping a Research Agenda. Canadian Journal of Nursing Research. 2014;46(3):26-45.
31. Crawford C, Teo L, Elfenbaum P, Enslein V, Deuster PA, Berry K. Methodological approach to moving nutritional science evidence into practice. Nutrition Reviews. 2017;75:6-16.
32. Cunningham RM, Carter PM, Ranney ML, Walton M, Zeoli AM, Alpern ER, et al. Prevention of Firearm Injuries Among Children and Adolescents: Consensus-Driven Research Agenda from the Firearm Safety Among Children and Teens (FACTS) Consortium. JAMA Pediatrics. 2019;173(8):780-9.
33. Dahabreh IJ, Chan JA, Earley A, Moorthy D, Avendano EE, Trikalinos TA, et al. Agency for Healthcare Research and Quality. 2017;16(17):01.
34. De Bortoli Cassiani SH, Bassalobre-Garcia A, ra, Reveiz L. Universal Access to Health and Universal Health Coverage: identification of nursing research priorities in Latin America. Revista Latino-Americana de Enfermagem (RLAE). 2015;23(6):1-14.
35. Deen J, Matos LdC, Temple B, Su J-Y, da Silva J, Liberato S, et al. Identifying national health research priorities in Timor-Leste through a scoping review of existing health data. Health Research Policy & Systems. 2013;11(1):8-.
36. Dilworth TJ, Pharm DS, Mott D, Young H. Pharmacists' communication with Spanish-speaking patients: a review of the literature to establish an agenda for future research. Research In Social & Administrative Pharmacy. 2009;5(2):108-20.
37. Effa EE, Oduwole O, Schoonees A, Hohlfeld A, Durao S, Kredo T, et al. Priority setting for new systematic reviews: processes and lessons learned in three regions in Africa. BMJ Global Health. 2019;4(4):e001615.
38. Fackrell K, Potgieter I, Shekhawat GS, Baguley DM, Sereda M, Hoare DJ. Clinical Interventions for Hyperacusis in Adults: A Scoping Review to Assess the Current Position and Determine Priorities for Research. BioMed Research International. 2017;2017:1-22.
39. Fadlallah R, Bou-Karroum L, El-Jardali F, Hishi L, Al-Akkawi A, Tsolakian IG, et al. Quality, safety and performance management in primary health care: from scoping review to research priority setting and implementation plan in the Eastern Mediterranean Region. BMJ Global Health. 2019;4:e001477.
40. Frutos Perez-Surio A, Gimeno-Gracia M, Alcacera Lopez MA, Sagredo Samanes MA, Pardo Jario MDP, Salvador Gomez MDT. Systematic review for the development of a pharmaceutical and medical products prioritization framework. Journal of Pharmaceutical Policy & Practice. 2019;12:21.
41. Fuentes JC, Cañón LA, Pérez ÁV, Pinzón CE, Pérez AM, Avellaneda PA, et al. Metodologías para la priorización en investigación en salud: una revisión sistemática de la literatura. Revista Panamericana de Salud Publica. 2017;41:1-8.
42. Ghijben P, Gu Y, Lancsar E, Zavarsek S. Revealed and Stated Preferences of Decision Makers for Priority Setting in Health Technology Assessment: A Systematic Review. PharmacoEconomics. 2018;36(3):323-40.
43. Goldstein A, Venker E, Chunhua W, Weng C. Evidence appraisal: a scoping review, conceptual framework, and research agenda. Journal of the American Medical Informatics Association. 2017;24(6):1192-203.
44. Goyet S, Touch S, Ir P, SamAn S, Fassier T, Frutos R, et al. Gaps between research and public health priorities in low income countries: evidence from a systematic literature review focused on Cambodia. Implementation Science. 2015;10(1):217-.
45. Greenhalgh T, Hinton L, Finlay T, Macfarlane A, Fahy N, Clyde B, et al. Frameworks for supporting patient and public involvement in research: Systematic review and co-design pilot. Health Expectations. 2019;22(4):785-801.
46. Grundy M, Ghazi F. Research priorities in haemato-oncology nursing: results of a literature review and a Delphi study. European Journal of Oncology Nursing. 2009;13(4):235-49.
47. Gu Y, Lancsar E, Ghijben P, Butler JR, Donaldson C. Attributes and weights in health care priority setting: A systematic review of what counts and to what extent. Social Science & Medicine. 2015;146:41-52.
48. Hamilton J, Abdiwahab E, Edwards H, Fang M-L, Jdayani A, Breslau E, et al. Primary care providers' cancer genetic testing-related knowledge, attitudes, and communication behaviors: A systematic review and research agenda. JGIM: Journal of General Internal Medicine. 2017;32(3):315-24.
49. Hartmann M, Khosla R, Krishnan S, George A, Gruskin S, Amin A. How Are Gender Equality and Human Rights Interventions Included in Sexual and Reproductive Health Programmes and Policies: A Systematic Review of Existing Research Foci and Gaps. PLoS ONE [Electronic Resource]. 2016;11(12):e0167542.
50. Hendry C, Walker A. Priority setting in clinical nursing practice: literature review. Journal of Advanced Nursing (Wiley-Blackwell). 2004;47(4):427-36.
51. Henrotin Y, Chevalier X, Herrero-Beaumont G, McAlindon T, Mobasheri A, Pavelka K, et al. Physiological effects of oral glucosamine on joint health: current status and consensus on future research priorities. BMC Research Notes. 2013;6:115.
52. Hertenstein E, Tang NKY, Bernstein CJ, Nissen C, Underwood MR, hu HK. Sleep in patients with primary dystonia: A systematic review on the state of research and perspectives. Sleep Medicine Reviews. 2016;26:95-107.
53. Hill SR, Vale L, Hunter D, Henderson E, Oluboyede Y. Economic evaluations of alcohol prevention interventions: Is the evidence sufficient? A review of methodological challenges. Health Policy. 2017;121(12):1249-62.
54. Hilliard TM, Boulton ML. Public health workforce research in review: a 25-year retrospective. American Journal of Preventive Medicine. 2012;42(5):S17-28.
55. Hoenig H, Siebens H. Research agenda for geriatric rehabilitation. American Journal of Physical Medicine & Rehabilitation. 2004;83(11):858-66.
56. Hsueh L, Hirsh AT, Maupome G, Stewart JC. Patient-Provider Language Concordance and Health Outcomes: A Systematic Review, Evidence Map, and Research Agenda. Medical Care Research & Review. 2019:1077558719860708.
57. Husereau D, Boucher M, Noorani H. Priority setting for health technology assessment at CADTH. International Journal of Technology Assessment in Health Care. 2010;26(3):341-7.
58. Jacobs E, Chen AH, Karliner LS, Agger-Gupta N, Mutha S. The need for more research on language barriers in health care: a proposed research agenda. Milbank Quarterly. 2006;84(1):111-33.
59. Jones C, Taylor J, MacKay K, Soliman F, Clayton E, Gadda AM, et al. The Landscape of UK Child Protection Research 2010 to 2014: A Mapping Review of Substantive Topics, Maltreatment Types and Research Designs. Child Abuse Review. 2017;26(1):8-18.
60. Jun M, Manns B, Laupacis A, Manns L, Rehal B, Crowe S, et al. Assessing the extent to which current clinical research is consistent with patient priorities: a scoping review using a case study in patients on or nearing dialysis. Canadian Journal of Kidney Health & Disease. 2015;2:35.
61. Kalaitzis IS, Rowbotham NJ, Smith SJ, Smyth AR. Do current clinical trials in cystic fibrosis match the priorities of patients and clinicans? A systematic review. Journal of Cystic Fibrosis. 2019;21:21.
62. Karimkhani C, Trikha R, Aksut B, Jones T, Boyers LN, Schlichte M, et al. Identifying gaps for research prioritisation: Global burden of external causes of injury as reflected in the Cochrane Database of Systematic Reviews. Injury. 2016;47(5):1151-7.
63. Knox K, Parkinson J, Pang B, Fujihira H, David P, Rundle-Thiele S. A Systematic Literature Review and Research Agenda for Organ Donation Decision Communication. Progress in Transplantation. 2017;27(3):309-20.
64. Ko C, Ma J, Bartnik R, Haney MH, Kang M. Ethical Leadership: An Integrative Review and Future Research Agenda. Ethics & Behavior. 2018;28(2):104-32.
65. Kong G, Creamer MR, Simon P, Cavallo DA, Ross JC, Hinds JT, et al. Systematic review of cigars, cigarillos, and little cigars among adolescents: Setting research agenda to inform tobacco control policy. Addictive Behaviors. 2019;96:192-7.
66. Langford R, Bonell C, Komro K, Murphy S, Magnus D, Waters E, et al. The Health Promoting Schools Framework: Known Unknowns and an Agenda for Future Research. Health Education & Behavior. 2017;44(3):463-75.
67. Le Reste JY, Nabbe P, Lingner H, Kasuba Lazic D, Assenova R, Munoz M, et al. What research agenda could be generated from the European General Practice Research Network concept of Multimorbidity in Family Practice? BMC Family Practice. 2015;16(1):1-8.
68. Le Reste JY, Nabbe P, Lingner H, Kasuba Lazic D, Assenova R, Munoz M, et al. What research agenda could be generated from the European General Practice Research Network concept of Multimorbidity in Family Practice? BMC Family Practice. 2015;16:125.
69. Lee K, Eckhardt J, Holden C. Tobacco industry globalization and global health governance: towards an interdisciplinary research agenda. Palgrave Communications. 2016;2.
70. Legg L, Pollock A, Langhorne P, Sellars C. A multidisciplinary research agenda for stroke rehabilitation. British Journal of Therapy & Rehabilitation. 2000;7(7):319-24.
71. Li T, Vedula SS, Scherer R, Dickersin K. What comparative effectiveness research is needed? A framework for using guidelines and systematic reviews to identify evidence gaps and research priorities. Annals of Internal Medicine. 2012;156(5):367-77.
72. Litchman ML, Schlepko T, Rowley T, McFarl, M, Fi, et al. A scoping review of Advanced Practice Registered Nurses Consensus Model outcomes: Part four of a four-part series on critical topics identified by the 2015 Nurse Practitioner Research Agenda. Journal of the American Association of Nurse Practitioners. 2018;30(12):710-23.
73. Maglione MA, Gans D, Das L, Timbie J, Kasari C. Nonmedical Interventions for Children With ASD: Recommended Guidelines and Further Research Needs. Pediatrics. 2012;130:S169-78.
74. Manikam L, Shah R, Reed K, Santini G, Lakhanpaul M. Using a co-production prioritization exercise involving South Asian children, young people and their families to identify health priorities requiring further research and public awareness. Health Expectations. 2017;20(5):852-61.
75. Maple M, Pearce T, Sanford R, Cerel J, Castelli Dransart DA, Andriessen K. A Systematic Mapping of Suicide Bereavement and Postvention Research and a Proposed Strategic Research Agenda. Crisis: Journal of Crisis Intervention & Suicide. 2018;39(4):275-82.
76. Marrie RA, Miller A, Sormani MP, Thompson A, Waubant E, Trojano M, et al. Recommendations for observational studies of comorbidity in multiple sclerosis. Neurology. 2016;86(15):1446-53.
77. Maxwell LJ, Wells GA, Simon LS, Conaghan PG, Grosskleg S, Scrivens K, et al. Current State of Reporting Pain Outcomes in Cochrane Reviews of Chronic Musculoskeletal Pain Conditions and Considerations for an OMERACT Research Agenda. Journal of Rheumatology. 2015;42(10):1934-42.
78. McGain F, Naylor C. Environmental sustainability in hospitals - a systematic review and research agenda. Journal of Health Services & Research Policy. 2014;19(4):245-52.
79. Mercadamte S. Death rattle: critical review and research agenda. Supportive Care in Cancer. 2014;22(2):571-5.
80. Meredith SE, Juliano LM, Hughes JR, Griffiths RR. Caffeine Use Disorder: A Comprehensive Review and Research Agenda. Journal of Caffeine Research. 2013;3(3):114-30.
81. Mirzoev T, Kane S. Key strategies to improve systems for managing patient complaints within health facilities - what can we learn from the existing literature? Global Health Action. 2018;11(1):1-.
82. Mitton C, Smith N, Peacock S, Evoy B, Abelson J. Public participation in health care priority setting: A scoping review. Health Policy. 2009;91(3):219-28.
83. Mobinizadeh M, Raeissi P, Nasiripour AA, Olyaeemanesh A, Tabibi SJ. The health systems' priority setting criteria for selecting health technologies: A systematic review of the current evidence. Medical Journal of the Islamic Republic of Iran. 2016;30:329.
84. Montesanti S, Robinson-Vollman A, Green LA. Designing a framework for primary health care research in Canada: a scoping literature review. BMC Family Practice. 2018;19(1):N.PAG-N.PAG.
85. Moos MK, Bartholomew NE, Lohr KN. Counseling in the clinical setting to prevent unintended pregnancy: an evidence-based research agenda. Contraception. 2003;67(2):115-32.
86. Morton MJ, DeAugustinis ML, Velasquez CA, Singh S, Kelen GD. Developments in Surge Research Priorities: A Systematic Review of the Literature Following the Academic Emergency Medicine Consensus Conference, 2007-2015. Academic Emergency Medicine. 2015;22(11):1235-52.
87. Murray SF, Pearson SC. Maternity referral systems in developing countries: current knowledge and future research needs. Social Science & Medicine. 2006;62(9):2205-15.
88. Nasser M, Ueffing E, Welch V, Tugwell P. An equity lens can ensure an equity-oriented approach to agenda setting and priority setting of Cochrane Reviews. Journal of Clinical Epidemiology. 2013;66(5):511-21.
89. Nicolau I, Ling D, Tian L, Lienhardt C, Pai M. Research questions and priorities for tuberculosis: a survey of published systematic reviews and meta-analyses. PLoS ONE [Electronic Resource]. 2012;7(7):e42479.
90. Nielsen CP, Funch TM, Kristensen FB. Health technology assessment: research trends and future priorities in Europe. Journal of Health Services Research & Policy. 2011;16:6-15.
91. Nielsen MB, Einarsen SV. What we know, what we do not know, and what we should and could have known about workplace bullying: An overview of the literature and agenda for future research. Aggression & Violent Behavior. 2018;42:71-83.
92. Nilsen ES, Myrhaug HT, Johansen M, Oliver S, Oxman AD. Methods of consumer involvement in developing healthcare policy and research, clinical practice guidelines and patient information material. Cochrane Database of Systematic Reviews. 2006:N.PAG-N.PAG.
93. Nyanchoka L, Tudur-Smith C, Thu VN, Iversen V, Tricco AC, Porcher R. A scoping review describes methods used to identify, prioritize and display gaps in health research. Journal of Clinical Epidemiology. 2019;109:99-110.
94. Odgers HL, Tong A, Lopez-Vargas P, Davidson A, Jaffe A, McKenzie A, et al. Research priority setting in childhood chronic disease: a systematic review. Archives of Disease in Childhood. 2018;103(10):942-51.
95. Odone A, Matteelli A, Chiesa V, Cella P, Ferrari A, Pezzetti F, et al. Assessing the impact of defining a global priority research agenda to address HIV-associated tuberculosis. Tropical Medicine & International Health. 2016;21(11):1420-7.
96. Okamoto I, Wright D, Foster C. Impact of cancer on everyday life: a systematic appraisal of the research evidence. Health Expectations. 2012;15(1):97-111.
97. Okl, T, Karimkhani C, Pederson H, Boyers LN, Sawyer MD, et al. Research prioritization of men's health and urologic diseases. International Braz J Urol. 2017;43(2):289-303.
98. Olding M, McMillan SE, Reeves S, Schmitt MH, Puntillo K, Kitto S. Patient and family involvement in adult critical and intensive care settings: a scoping review. Health Expectations. 2016;19(6):1183-202.
99. oll HH, Madhok R. From evidence to best practice in the management of fractures of the distal radius in adults: working towards a research agenda. BMC Musculoskeletal Disorders. 2003;4:27.
100. Osthoff A-KR, Niedermann K, Braun J, Adams J, Brodin N, Dagfinrud H, et al. 2018 EULAR recommendations for physical activity in people with inflammatory arthritis and osteoarthritis. Annals of the Rheumatic Diseases. 2018;77(9):1251-60.
101. Otto JL, Beech EH, Evatt DP, Belsher BE, Workman DE, Campbell MS. A systematic approach to the identification and prioritization of psychological health research gaps in the Department of Defense. Military Psychology (American Psychological Association). 2018;30(6):557-63.
102. Parker JA, Barroso F, Stanworth SJ, Spiby H, Hopewell S, Doree CJ, et al. Gaps in the evidence for prevention and treatment of maternal anaemia: a review of systematic reviews. BMC Pregnancy & Childbirth. 2012;12:56.
103. Pebsworth PA, Huffman MA, Lambert JE, Young SL. Geophagy among nonhuman primates: A systematic review of current knowledge and suggestions for future directions. American Journal of Physical Anthropology. 2019;168:164-94.
104. Pederson H, Okl, T, Boyers LN, Karimkhani C, Rosenfeld RM, et al. Identifying otolaryngology systematic review research gaps: comparing Global Burden of Disease 2010 results with Cochrane Database of Systematic Review content. JAMA Otolaryngology-- Head & Neck Surgery. 2015;141(1):67-72.
105. Penakalapati G, Swarthout J, Delahoy MJ, McAliley L, Wodnik B, Levy K, et al. Exposure to Animal Feces and Human Health: A Systematic Review and Proposed Research Priorities. Environmental Science & Technology. 2017;51(20):11537-52.
106. Pescatello LS, MacDonald HV, Ash GI, Lamberti LM, Farquhar WB, Arena R, et al. Assessing the Existing Professional Exercise Recommendations for Hypertension: A Review and Recommendations for Future Research Priorities. Mayo Clinic Proceedings. 2015;90(6):801-12.
107. Peterson K, Anderson J, Boundy E, Ferguson L, McCleery E, Waldrip K. Mortality Disparities in Racial/Ethnic Minority Groups in the Veterans Health Administration: An Evidence Review and Map...See also Ibrahim, p. 299. American Journal of Public Health. 2018;108(3):e1-e11.
108. Petkovic J, Barton JL, Flurey C, Goel N, Bartels CM, Barnabe C, et al. Health Equity Considerations for Developing and Reporting Patient-reported Outcomes in Clinical Trials: A Report from the OMERACT Equity Special Interest Group. Journal of Rheumatology. 2017;44(11):1727-33.
109. Pierce JS, Lacey SE, Lippert JF, Lopez R, Franke JE. Laser-Generated Air Contaminants from Medical Laser Applications: A State-of-the-Science Review of Exposure Characterization, Health Effects, and Control. Journal of Occupational & Environmental Hygiene. 2011;8(7):447-66.
110. Piga M, Cangemi I, Mathieu A, ro, Cauli A. Telemedicine for patients with rheumatic diseases: Systematic review and proposal for research agenda. Seminars in Arthritis & Rheumatism. 2017;47(1):121-8.
111. Plusnin N, Pepping CA, Kashima ES. The Role of Close Relationships in Terror Management: A Systematic Review and Research Agenda. Personality & Social Psychology Review. 2018;22(4):307-46.
112. Potgieter I, MacDonald C, Partridge L, Cima R, Sheldrake J, Hoare DJ. Misophonia: A scoping review of research. Journal of Clinical Psychology. 2019;75(7):1203-18.
113. Pozzar RA, Berry DL. Patient-centered research priorities in ovarian cancer: A systematic review of potential determinants of guideline care. Gynecologic Oncology. 2017;147(3):714-22.
114. Price A, Albarqouni L, Kirkpatrick J, Clarke M, Liew SM, Roberts N, et al. Patient and public involvement in the design of clinical trials: An overview of systematic reviews. Journal of Evaluation in Clinical Practice. 2018;24(1):240-53.
115. Ranson K, Law TJ, Bennett S. Establishing health systems financing research priorities in developing countries using a participatory methodology. Social Science & Medicine. 2010;70(12):1933-42.
116. Ravelli A, Consolaro A, ro, Horneff G, Laxer RM, Lovell DJ, et al. Treating juvenile idiopathic arthritis to target: recommendations of an international task force. Annals of the Rheumatic Diseases. 2018;77(6):819-28.
117. Richter S, Chaw-Kant J. Canadian homeless women: gaps in the research agenda. International Journal of Child Health & Human Development. 2010;3(1):7-15.
118. Riffin C, Pillemer K, Chen EK, Warmington M, Adelman RD, Reid MC. Identifying Key Priorities for Future Palliative Care Research Using an Innovative Analytic Approach. American Journal of Public Health. 2015;105(1):e15-21.
119. Robert G, Milne R. A Delphi study to establish national cost-effectiveness research priorities for positron emission tomography. European Journal of Radiology. 1999;30(1):54-60.
120. Rowbotham NJ, Smith S, Prayle AP, Robinson KA, Smyth AR. Gaps in the evidence for treatment decisions in cystic fibrosis: a systematic review. Thorax. 2019;74(3):229-36.
121. Sbaffi L, Rowley J. Trust and Credibility in Web-Based Health Information: A Review and Agenda for Future Research. Journal of Medical Internet Research. 2017;19(6):1-.
122. Shattuck PT, Lau L, Anderson KA, Kuo AA. A National Research Agenda for the Transition of Youth With Autism. Pediatrics. 2018;141:S355-S61.
123. Sigfrid L, Moore C, Salam AP, Maayan N, Hamel C, yce, et al. A rapid research needs appraisal methodology to identify evidence gaps to inform clinical research priorities in response to outbreaks-results from the Lassa fever pilot. BMC Medicine. 2019;17(1):1-17.
124. Simonetti M, Aiken LH, Lake ET. Nursing in Chilean Hospitals: A Research Agenda to Inform Health Policies and Improve Patient Outcomes. Hispanic Health Care International. 2019;17(2):79-88.
125. Singh AS, Saliasi E, van den Berg V, Uijtdewilligen L, de Groot RHM, Jolles J, et al. Effects of physical activity interventions on cognitive and academic performance in children and adolescents: a novel combination of a systematic review and recommendations from an expert panel. British Journal of Sports Medicine. 2019;53(10):640-7.
126. Stewart RJ, Caird J, Oliver K, Oliver S. Patients' and clinicians' research priorities. Health Expectations. 2011;14(4):439-48.
127. Story WT. Social capital and health in the least developed countries: A critical review of the literature and implications for a future research agenda. Global Public Health. 2013;8(9):983-99.
128. Swingler GH, Irlam JH, Macharia WM, Tietche F, Meremikwu MM. A systematic review of existing national priorities for child health research in sub-Saharan Africa. Health Research Policy & Systems. 2005;3:7.
129. Synnot AJ, Tong A, Bragge P, Lowe D, Nunn JS, O'Sullivan M, et al. Selecting, refining and identifying priority Cochrane Reviews in health communication and participation in partnership with consumers and other stakeholders. Health Research Policy & Systems. 2019;17(1):N.PAG-N.PAG.
130. Tempfer CB, Nowak P. Consumer participation and organizational development in health care: a systematic review. Wiener Klinische Wochenschrift. 2011;123(13):408-14.
131. Thome AMT, Ceryno PS, Scavarda A, Remmen A. Sustainable infrastructure: A review and a research agenda. Journal of Environmental Management. 2016;184:143-56.
132. Turner L, Calvert HG. The Academic, Behavioral, and Health Influence of Summer Child Nutrition Programs: A Narrative Review and Proposed Research and Policy Agenda. Journal of the Academy of Nutrition & Dietetics. 2019;119(6):972-83.
133. Varker T, Metcalf O, Forbes D, Chisolm K, Harvey S, Van Hooff M, et al. Research into Australian emergency services personnel mental health and wellbeing: An evidence map. Australian & New Zealand Journal of Psychiatry. 2018;52(2):129-48.
134. Vat LE, Finlay T, Jan Schuitmaker-Warnaar T, Fahy N, Robinson P, Boudes M, et al. Evaluating the "return on patient engagement initiatives" in medicines research and development: A literature review. Health Expectations. 2019;6:06.
135. Vorkoper S, Kupfer LE, An, N, Patel P, Beecroft B, et al. Building on the HIV chronic care platform to address noncommunicable diseases in sub-Saharan Africa: a research agenda. AIDS (02699370). 2018;32:S107-S13.
136. Votruba N, Ziemann A, ra, Grant J, Thornicroft G. A systematic review of frameworks for the interrelationships of mental health evidence and policy in low- and middle-income countries. Health Research Policy & Systems. 2018;16(1):N.PAG-N.PAG.
137. Wald HL, Leykum LK, Mattison ML, Vasilevskis EE, Meltzer DO. Road map to a patient-centered research agenda at the intersection of hospital medicine and geriatric medicine. Journal of General Internal Medicine. 2014;29(6):926-31.
138. Watson SK, Rudge JW, Coker R. Health Systems' 'Surge Capacity': State of the Art and Priorities for Future Research. Milbank Quarterly. 2013;91(1):78-122.
139. Wolf FM, Shea JA, Albanese MA. Toward setting a research agenda for systematic reviews of evidence of the effects of medical education. Teaching & Learning in Medicine. 2001;13(1):54-60.
140. Yamey G, Horváth H, Schmidt L, Myers J, Brindis CD. Reducing the global burden of Preterm Birth through knowledge transfer and exchange: a research agenda for engaging effectively with policymakers. Reproductive Health. 2016;13:1-9.
